# Purification and Characterization of a Novel Intracellular Sucrase Enzyme of* Leishmania donovani* Promastigotes

**DOI:** 10.1155/2016/7108261

**Published:** 2016-04-14

**Authors:** Arpita Singh, Debjani Mandal

**Affiliations:** Division of Infectious Diseases and Immunology, Indian Institute of Chemical Biology, 4 Raja S.C. Mullick Road, Jadavpur, Kolkata, West Bengal 700032, India

## Abstract

The promastigote stage of* Leishmania* resides in the sand fly gut, enriched with sugar molecules. Recently we reported that* Leishmania donovani* possesses a sucrose uptake system and a stable pool of intracellular sucrose metabolizing enzyme. In the present study, we purified the intracellular sucrase nearly to its homogeneity and compared it with the purified extracellular sucrase. The estimated size of intracellular sucrase is ~112 kDa by gel filtration chromatography, native PAGE, and substrate staining. However, in SDS-PAGE, the protein is resolved at ~56 kDa, indicating the possibility of a homodimer in its native state. The kinetics of purified intracellular sucrase shows its higher substrate affinity with a *K*_*m*_ of 1.61 mM than the extracellular form having a *K*_*m*_ of 4.4 mM. The highly specific activity of intracellular sucrase towards sucrose is optimal at pH 6.0 and at 30°C. In this report the purification and characterization of intracellular sucrase provide evidence that sucrase enzyme exists at least in two different forms in* Leishmania donovani* promastigotes. This intracellular sucrase may support further intracellular utilization of transported sucrose.

## 1. Introduction

Sucrose, glucose, and other hexose's are required for the maintenance of parasite redox balance and for generating precursors for DNA and RNA biosynthesis. As a result* Leishmania donovani* depends on carbohydrates to sustain their central carbon metabolism. This parasite has the ability to modify its biochemical machinery to adapt diverse microenvironment, encountered within the host in order to guarantee their survival.

Sugar meal is important for the development of infective forms of* Leishmania* sp. and for its virulency [1, 2]. Considering the major metabolite constituents in sandfly gut [3, 4], sucrose presumably is one of the preferred energy source where the division of the promastigotes takes place. Therefore it is important to understand how they utilize this sugar as well as the mechanism of sugar uptake inside the cell. Recently we have reported the sucrose transport system in* Leishmania donovani* promastigotes and the intracellular sucrose splitting enzyme sucrase, which may demonstrate the utilization of internalized sucrose [5]. However, sucrose internalization and henceforth consumption is a relatively unexplored area in* Leishmania* biology. Leishmaniasis still remains a major health concern of the 21st century throughout the world, despite the sustained efforts to control the disease over several decades. Along with existing efforts of developing vaccines [6] and improved drugs [7] it is necessary to understand its physiology foremost and the inherent ability of the parasite to adapt itself to a myriad of adverse environmental parameters.

Our recent finding on the intracellular pool of sucrase enzyme, as well as previous reports on secretary extracellular sucrase [5, 8], prompted us to purify the intracellular sucrase. Here we identified a ~112 kDa homodimer intracellular sucrase enzyme of* Leishmania donovani* promastigotes and characterized and compared it simultaneously with the purified ~71 kDa extracellular sucrase. Further detailed molecular characterization of the intracellular enzyme is important to gain insight into its probable role in biochemical pathway of the parasite and pathogenesis. This understanding may contribute knowledge towards antileishmanial drug designing.

## 2. Materials and Methods

### 2.1. Materials

Analytical grade reagents were used for experimental purpose. All the chemicals were purchased from Sigma, USA, unless otherwise mentioned. Brain Heart Infusion was obtained from Acumedia Manufacturers Inc. Baltimore, MD, USA, and Media 199, Penicillin-Streptomycin powder, were purchased from GIBCO, USA.

### 2.2. Strains

The strain of* L. donovani* used in this work, MOHM/IN/1978/UR6, was a clinical isolate from an Indian patient with confirmed Kala-azar collected in the year of 1978. UR6 cells were maintained in solid blood agar media of pH 7.4 and highly motile promastigotes were considered during experiments.

### 2.3. Media and Culture Conditions

#### 2.3.1. Solid Blood Agar Media

According to Kumar Saha et al. [9] the cell line was maintained in solid blood agar media at 22°C. The growth of promastigotes was measured every 24 hrs of 72 hrs of growth period.

#### 2.3.2. Liquid Media 199

One liter of liquid medium was prepared by adding 11 g of media-199 power, 10% FCS, 22 mM Hepes, and 100 units of Penicillin-Streptomycin in water. The pH of the media was adjusted to 7.4 and filter sterilized for further use.

### 2.4. Preparation of Cell-Free Extract

Cell-free extract was prepared according to Singh and Mandal [5]. Exponentially growing promastigotes in liquid culture were harvested, washed with PBS, and suspended in lysis buffer containing (5 mM Tris-HCl, 0.5 mM PMSF, and 0.25 mM EDTA, pH 7.4). The suspension incubated at ambient temperature was mixed 10 times by vortexing 30 secs at 2 min intervals. This was further sonicated at 15 pulses of 20 secs each with 1 min interval on ice. The sonicated extract was adjusted to 50 mM Tris-HCl, pH 7.4 (Buffer A), and ultracentrifuged at 1 × 10^5^ g for 1 hr at 4°C. The supernatant considered as cell-free extract was collected and stored at −20°C for further use.

### 2.5. Purification Procedure

#### 2.5.1. Enzyme Extraction

The enzyme activity of the cell free extract was considered as crude and its level of activity was taken to be 100% for calculation of recovery.

#### 2.5.2. Ammonium Sulfate Precipitation

Solid ammonium sulfate was added into the cell-free extract, first to 33% saturation and then to 75% saturation. The pellet holding major sucrase activity was finally suspended in Buffer A containing protease inhibitor and immediately considered for further purification.

#### 2.5.3. Size-Exclusion Chromatography (SEC)

Following ammonium sulfate concentration the resuspended sample (active fraction) was loaded onto a Sephacryl S-200 column (120 × 1 cm) preequilibrated with Buffer A. The proteins were eluted with Buffer A at a flow rate of 22 mL/hr. Protein fractions/tube containing the major activity of sucrase were pooled for further steps of purification.

#### 2.5.4. Ion-Exchange Chromatography (IEC)

A column (20 × 2 cm) was packed with CM-Cellulose matrix, swollen overnight at room temperature to have a bed volume of 10 × 2 cm. The pooled active enzyme fractions from S-200 column were passed through CM-Cellulose (cation exchanger) column preequilibrated with Buffer A for further purification. The flow-through containing the major sucrase activity was pooled immediately and subjected to DEAE Sephadex (anion exchanger) column of same bed volume preequilibrated with Buffer A. The bound protein containing the enzyme fraction was eluted with a salt gradient of 0–0.2 M NaCl. The pooled fraction from DEAE Sephadex was passed through S-200 column (120 × 1 cm) again to equilibrate the semipurified enzyme sample with 20 mM potassium-phosphate buffer pH 7.4 for Hydroxyapatite batch adsorption.

#### 2.5.5. Hydroxyapatite Batch Adsorption

Hydroxyapatite [10] matrix was equilibrated with 20 mM potassium-phosphate buffer at pH 7.4. The pooled fraction containing major sucrase activity from S-200 column was subjected to Hydroxyapatite batch adsorption and allowed the enzyme to mix properly by occasional stirring. After the matrix settled down, the unabsorbed content, mostly purified enzyme, was collected by a low spin centrifugation at 4°C. The supernatant was found to contain >95% purified enzyme.

### 2.6. Protein Estimation

Modified Lowry method was used for protein estimation [11] and BSA was taken as a standard.

### 2.7. Gel Electrophoresis and Activity Staining

Polyacrylamide gel electrophoresis (PAGE) under native and denaturing condition was performed according to Laemmli's discontinuous Tris-glycine buffer system [12] with little modification. During activity staining the gel was sliced into two halves, each half bearing identical samples. One part of 5.5% native gel containing the purified intracellular sucrase enzyme was incubated in 50 mM Tris-HCl with 100 mM sucrose for 2 hrs at 30°C followed by a wash with distilled water [13]. The gel was then immersed in 1 M of iodoacetamide for 15 min at room temperature. Following the wash with double distilled water, the gel was incubated in 0.5 N NaOH containing 2% 2,3,5-Triphenyl Tetrazolium Chloride (TTC) for 15 min in a boiling water bath until a diffuse pink background color develops. After proper distaining with 7.5% acetic acid, a photo was taken immediately as the color developed persists very shortly. The other gel part was subsequently stained in coomassie R-250 for the corresponding protein band identification.

### 2.8. Purification of Extracellular Sucrase

Promastigote cells grew in liquid culture media for 66-67 hrs were pelleted down by centrifugation and the cell-free media was lyophilized to semidryness. It was reconstituted in 50 mM Potassium-Phosphate buffer, pH 7.4, and concentrated to ~1.5 mL by using a centricon membrane filter YM 10. Following the same purification procedure of intracellular sucrase, the filtrate was allowed to pass through the preequilibrated S-200 column. Subsequent ion-exchange chromatography and Hydroxyapatite batch adsorption steps were performed to obtain purified extracellular enzyme.

### 2.9. Enzyme Assays

With slight modification to Messer and Dahlqvist's method [14] sucrase activity was estimated. The enzyme reaction was initiated by adding the enzyme in the assay mixture (50 mM sodium acetate, pH 5.5/6.0) in presence of 4 mM of substrate sucrose and the reaction stopped by heat inactivation after 30 min of incubation at 30°C. The colorimetric estimation of glucose was taken at 505 nm. One unit of enzyme activity is defined as the amount of enzyme that hydrolyzes sucrose to produce 1 mole of glucose at 30°C.

### 2.10. Optimum Temperature and Thermostability

The optimum temperature for the enzyme was evaluated by measuring sucrase activity in 50 mM sodium acetate buffer (pH 5.5–6.0) at different temperature. Thermostability was determined by preincubating the purified enzyme for 30 min at a range of temperature (4–60°C) prior to the standard activity assay.

### 2.11. Optimum pH

Purified enzyme activity was assayed in 50 mM of four different buffering agents, glycine-HCl (pH 2.0-3.0), acetate (pH 4.0–6.0), phosphate (pH 6.0-7.0), and Tris-HCl (pH 7.0-8.0), in order to record the pH profile under the standard experimental conditions.

### 2.12. Effect of Metal Ions on Intracellular Sucrase Activity

The metal ions effect on enzyme activity was determined after preincubating the purified enzyme with various metal ions such as ZnCl_2_, HgCl_2_, CaCl_2_, KCl, AgNO_3_, FeSO_4_, MgSO_4_, MnSO_4_, CuSO_4_, CoSO_4_, and NiSO_4_, one at a time at desired concentration for 10 min at 30°C. Following standard assay condition enzyme activity was measured and expressed as percentage of control (without metal ion).

## 3. Results

In the present study both the intra- and extracellular enzymes were simultaneously purified to characterize the intracellular sucrase enzyme in comparison to extracellular one. The molecular size of intracellular sucrase was confirmed by size-exclusion chromatography and native gel analysis followed by activity staining.

### 3.1. Molecular Weight Determination

#### 3.1.1. Size-Exclusion Chromatography

**Figure 1:**
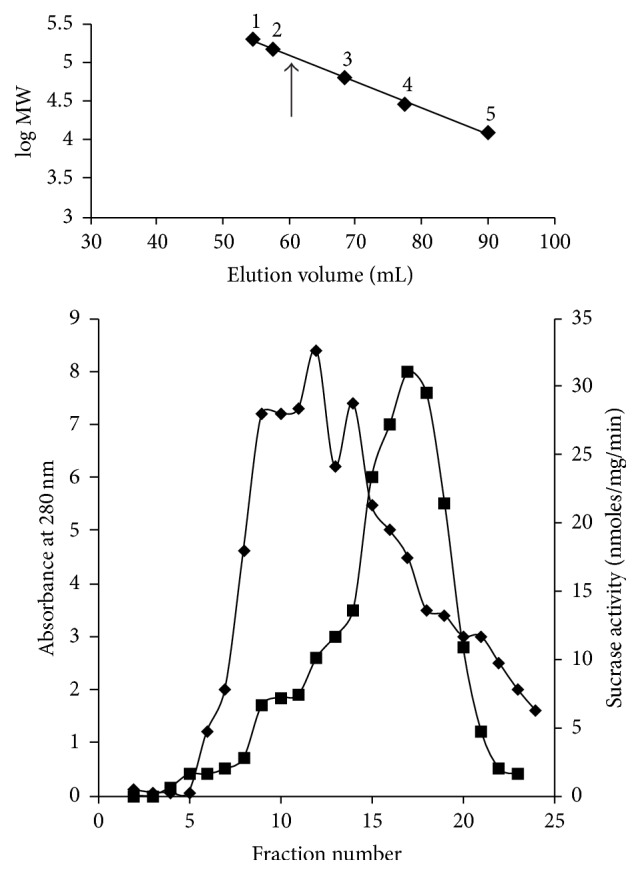
Size-exclusion chromatography of intracellular sucrase. The ammonium sulfate saturated sample protein was run on a Sephacryl (S-200) column, preequilibrated with Buffer A. The protein absorbance measured at 280 nm (-*◆*-) was plotted on primary axis. The secondary *y*-axis shows the intracellular sucrase activity (-■-) of the corresponding fractions. The molecular weight of the native enzyme was determined to be ~112 kDa from the S-200 calibration graph (Figure 1 inset). The ↑ arrow indicates the peak of intracellular sucrase elution volume and the molecular weight (MW) of the protein. The molecular weight markers used were as follows: amylase MW = 200,000; alcohol dehydrogenase MW = 150,000; hemoglobin MW = 64,500; carbonic anhydrase MW = 29,000; and cytochrome C MW = 12,400.

Size-exclusion chromatography of cell-free extract followed by ammonium sulfate precipitation shows intracellular sucrase activity (Figure 1) and the molecular mass of the protein estimated from gel filtration chromatography was approximately 112 kDa (Figure 1 inset). The active fractions from S-200 column were pooled and passed through different steps (Table 1) to get pure active protein. The entire purification steps yield nearly 330-fold the purified intracellular sucrase.

#### 3.1.2. Relative Mobility

**Figure 2:**
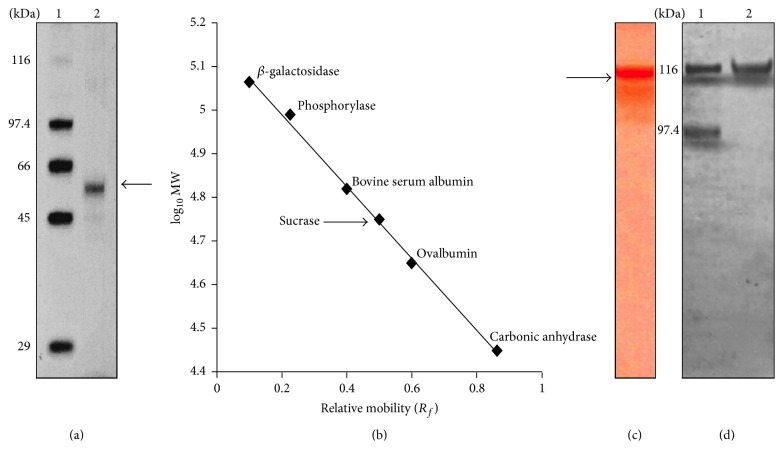
Gel electrophoretic analysis and activity staining of purified intracellular sucrase. (a) Electrophoresis (10% SDS-PAGE) was done in a discontinuous buffer system. Lane 1: molecular mass standards indicated on the left are as follows: galactosidase (116 kDa), phosphorylase B (97.4 kDa), bovine serum albumin (66 kDa), ovalbumin (45 kDa), carbonic anhydrase (29 kDa); lane 2 shows the purified intracellular sucrase protein band of nearly ~56 kDa in size. (b) 10% SDS-PAGE standard curve plotted with logarithm of the molecular weight against relative mobility. The arrow indicates the log of molecular weight of intracellular sucrase subunit in 10% SDS-PAGE. (c) Substrate staining of purified intracellular sucrase in native gel; the arrow indicates the activity band in native gel. (d) Coomassie stained purified intracellular sucrase in native gel. Lane 1 represents the proteins galactosidase (116 kDa) and phosphorylase B (97.4 kDa) as molecular weight marker and lane 2 shows the native form of purified intracellular sucrase.

Presence of a single band in SDS-PAGE (Figure 2(a)) reveals the purity of the enzyme. The estimated size of the protein is nearly 56 kDa as calculated from the relative mobility and the molecular weight of the known marker protein (Figure 2(b)). The 56 kDa protein size is nearly half the size estimated from gel filtration chromatography (112 kDa. Figure 1 inset), which may denote the homodimerization of the enzyme sucrase in its native state.

#### 3.1.3. Activity Staining

A confirmatory test for identifying the enzyme is to locate the enzymatic activity in the polyacrylamide gel. Thus, to confirm the position of intracellular sucrase amidst electrophoretically separated proteins, a modified approach of Gabriel and Wang [13] was used for substrate staining of the native intracellular sucrase. Purified protein shows a deep pinkish violet band, representing the active protein (Figure 2(c)). The subsequent single band in coomassie stain corresponding to the substrate stain (Figure 2(d)) confirms its approximate size of 112 kDa. This result corroborates with the protein size estimated from the gel filtration chromatography.

### 3.2. Enzyme Kinetics of Crude and Purified Intracellular Sucrase

**Figure 3:**
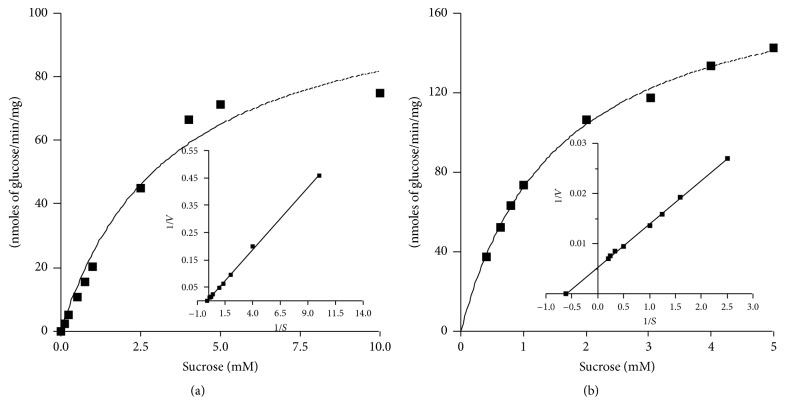
Enzyme kinetics of crude and purified intracellular sucrase: intracellular sucrase was incubated in the assay mixture with varying range of substrate concentration (0.20–10 mM) at a fixed incubation time to estimate the enzyme activity of crude (a) and purified (b) intracellular sucrase. The inset represents the Lineweaver-Burk plot of velocity and substrate concentration of crude and purified intracellular sucrase which shows *K*_*m*_ of 6.6 mM and 1.61 mM of sucrose, respectively. The results are the mean of three independent experiments (*n* = 3).

The intracellular sucrase activity was measured at different substrate concentration. The range of substrate concentration varied from 0.05–10 mM to 0.4–5.0 mM, respectively, for estimating the crude and purified intracellular sucrase enzyme activity. The Lineweaver-Burk plot of the crude intracellular sucrase enzyme activity shows the *K*_*m*_ of ~6.6 mM and the *V*_max_ of ~125 nmoles/min/mg (Figure 3(a)), while the purified intracellular enzyme has more affinity towards the substrate as calculated from the Lineweaver-Burk plot and the estimated *K*_*m*_ and *V*_max_ are ~1.6 mM and ~190.5 nmoles/min/mg, respectively (Figure 3(b)).

### 3.3. Substrate Specificity

Different disaccharides were used to check the substrate specificity of the purified intracellular enzyme. The enzyme was incubated in presence of different substrate, namely, raffinose, melibiose, maltose, trehalose, and palatinose, and the corresponding enzyme activity was measured according to Messer and Dahlqvist [14] in comparison with sucrose as a control (100%). The substrate specificity of the* Leishmania* intracellular sucrase is highly specific in nature, as it was unable to hydrolyze any of the substrates mentioned above except sucrose and partially raffinose at a concentration range from 1 to 10 mm (data not shown).

### 3.4. Molecular Mass and Kinetics of Extracellular Sucrase

Molecular mass of extracellular sucrase, determined by size-exclusion chromatography and SDS-PAGE (data not shown), was ~70.79 kDa. For kinetic study, enzyme activity of the purified extracellular sucrase was estimated by incubating with varying substrate concentration (0.125–8 mM) at 30°C for 30 min. The double reciprocal plot of the velocity of the reaction and the substrate shows the *K*_*m*_ of purified extracellular sucrase as ~4.4 mM, which corroborates the report on purified extracellular sucrase of* Leishmania* by Gontijo et al. [15]. The result illustrates that the purified extracellular sucrase has nearly three times reduced substrate affinity than that of intracellular sucrase enzyme.

Purification and the subsequent kinetic studies of the purified enzyme further confirmed that at least two different forms of the enzyme sucrase exist in* L. donovani* promastigotes.

### 3.5. Temperature Tolerance and pH Sensitivity of Intra- and Extracellular Sucrase

**Figure 4:**
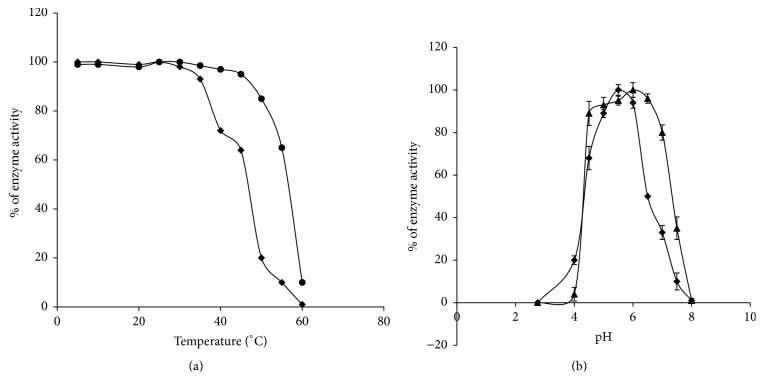
The temperature tolerance and optimum pH of intra- and extracellular sucrase: (a) the intra- and extracellular enzymes were preincubated for 30 min at different temperature (4–60°C) prior to estimating the enzyme activity under standard assay condition in 50 mM sodium acetate buffer at pH 6.0 and pH 5.5, respectively. The enzyme activities of intracellular (-*◆*-) and extracellular sucrase (-●-) were plotted as percentage of maximum activity (100%) at different temperature. (b) The pH optimum was measured by performing activity assay at different pH at a range of 02.75–8.0 as mentioned in Materials and Methods. The intracellular (-▲-) and extracellular (-*◆*-) sucrase activities were plotted as a percentage of activity versus pH, with maximum activity being 100%. Each point represents the mean of three different experiments.

Intracellular sucrase is susceptible to higher temperatures and loses 50% of its activity with an increase of temperature above 45°C. On the other hand the extracellular sucrase is mostly stable up to 50°C and has a wide range of temperature tolerance (Figure 4(a)). The intracellular sucrase shows ~20% of its maximum activity at this temperature then gets deactivated completely with further increase of temperature. However both the purified enzymes exhibit its maximum activity at 30°C (data not shown).

Interestingly the intracellular sucrase shows nearly 80% of its activity at a pH range from 4.5 to 7.0 (Figure 4(b)); nevertheless, the optimum enzyme activity occurs at pH 6.0 (data not shown). In standard assay condition the maximum enzyme activity of purified extracellular sucrase appears at pH 5.5 (data not shown) although more than 80% of its activity was observed between pH 5.0 and 6.5 (Figure 4(b)).

### 3.6. Effect of Metal Ion on Intracellular Sucrase Activity

**Figure 5:**
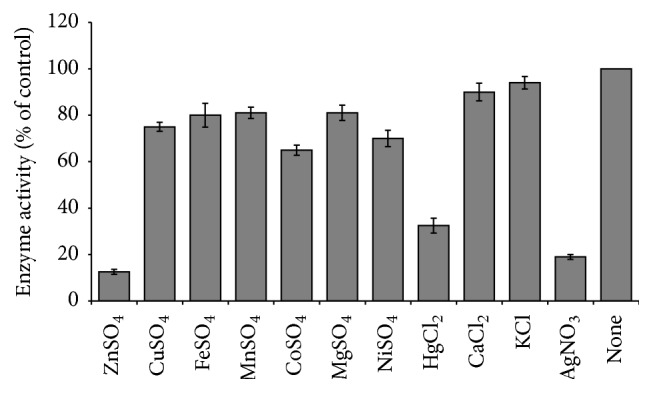
Effect of metal ion on the intracellular sucrase enzyme activity. The purified enzyme was preincubated with metal ions for 10 min at room temperature and the activity assay was performed in 50 mM sodium acetate buffer at pH 6.0. The enzyme activity in absence of metal ions was considered to be 100% and compared the relative activity of the enzyme in presence of different metal ions. The value presented corresponds to the mean value of three replicates.

The enzyme activity was compared in presence of various metal ions at 1 mM concentration as presented in Figure 5. Metal ions such as ZnCl_2_, AgNO_3_, and HgCl_2_ strongly inhibited the enzyme activity, while a moderate inhibition of activity was noted by the sulfate of cobalt, nickel, and copper ions.

## 4. Discussion

The parasites in the insect vector are exposed to an entirely different microenvironment than their vertebrate host.* Leishmania* promastigotes possess sucrose transporter in the plasma membrane [5], which helps the internalization of sucrose, a major food constituent of the insect gut. To address the issue on further utilization of the accumulated sucrose in* L. donovani* promastigotes we focus on the sucrose metabolizing enzyme sucrase. Recently we reported [5] that the majority of enzyme remains inside the cell as intracellular sucrase and the rest is secreted as extracellular sucrase. These sucrase enzymes are constitutive in nature; the specific activity of the enzyme remains the same in the presence or absence of external pressure (i.e., sucrose) in the media. The *K*_*m*_ of intracellular sucrase in cell-free extract and the purified form differs markedly from each other. Reduced affinity of the crude enzyme towards the substrate may occur by the interference of cytosolic inhibitory factors.

The distinctive characteristic properties of the enzyme sucrase suggest the possibility of having two forms of enzyme in* Leishmania* promastigotes, a ~71 kDa monomer extracellular sucrase and a ~56 kDa homodimer of intracellular sucrase. So there is a probability that the monomer of intracellular sucrase with an active catalytic site may be posttranslationally modified to become extracellular sucrase [8]. However, to determine whether each monomer is catalytically active or not, further studies need to be done. The significant difference observed in the kinetics between the two forms of the sucrase may be due to differing accessibility and efficiency of catalytic sites.

It has been established that the two forms of invertase extracellular and intracellular one in* Saccharomyces cerevisiae*, do not differ much in *K*_*m*_ or velocity, yet pH stability changes [16]. According to Wallis et al. [17]* Aspergillus niger* secretes two fructofuranosidases and both may be dimers in their natural conformation considering the protein size in SDS-PAGE and native state. The enzymes have affinity towards sucrose and to some extent to raffinose; however, their affinity varies with other substrates. In* Leishmania* the wide range of temperature tolerance of extracellular sucrase is probably to remain functional in outside temperatures; in contrast the intracellular sucrase has slight temperature tolerance (Figure 4(a)). Strong inhibition of intracellular enzyme activity with thiol modifying reagents like 5,5′-dithiobis-2-nitrobenzoic acid (DTNB), N-ethylmaleimide (NEM), and p-chloromercuribenzoate (PCMB) suggests that the active site of the enzyme may possess –SH moieties (data not shown). This finding corroborates the report on purified b-fructofuranosidase of* B. infantis* [18].

The enzymatic characterization and preliminary data on the N terminal sequence of the purified intracellular sucrase show its considerable homology with glycosidase, the bacterial *β*-fructofuranosidase class of enzyme [19], under the broader heading glycosidase (Singh and Mandal, unpublished data). This supports the findings of bacterial nature in many of the enzymes, which lies in the metabolic machinery of* Leishmania* [20]. The *β*-fructofuranosidases belong to the glycosyl hydrolase's family of 32 proteins and catalyze the hydrolysis of sucrose to glucose and fructose. Literature survey suggests that the bifidobacterial *β*-fructofuranosidases also have activity against the longer chained substrates such as raftilose, raftiline, and inulin [21]. The phylogenetic analysis of the related intracellular fructofuranosidases includes a large group of sucrose-6P-hydrolases, of which all are physically linked with genes encoding sucrose transport proteins of the PTS [20]. Very recently Lyda et al. [22] discovered the secretory invertase (LdINV) gene of* Leishmania* promastigotes and also identified a beta-fructofuranosidase-like gene, during the homology search of LdINV, which encodes a 120 kDa protein. To validate our preliminary results further study is necessary to identify the gene encoding intracellular sucrase.

The uptake and subsequent metabolism of glucose in Trypanosomatidae is an example of an adjustment leading to maximum energy efficiency [23]. However, it varies from species to species as* L. donovani* is confronted with widely varying conditions in the sandfly gut and strives for internal homeostasis even at the expense of energy. Thus it may happen that two different metabolic strategies represent two opposing trends: the capability of uptake of sucrose and its utilization by hydrolyzing sucrose to glucose and fructose are the efficient adaptation at the expense of short term flexibility and on the other hand the ability to rapidly adapt to environmental changes at the expense of energy.

Interestingly very recently Dirkx et al. [24] reported that the flagellated protozoan* Trichomonas vaginalis* genome contains nearly 11 putative sucrose transporters and a putative ß-fructofuranosidase (invertase). Thus, the machinery for both uptake and cleavage of intracellular sucrose appears to be present in the protozoa as the cell lysates retain invertase activity. It is likely that the most recent common ancestor of* T. vaginalis* was a gut-dwelling protist, where the capacity to utilize fructose containing compounds might be advantageous [25].

Representatives of the Kinetoplastids spread over a variety of different environments and, in due time, many of them became parasites of insects, leeches, major vertebrates lineages, and even plants. Where Kinetoplastids evolved to digenetic parasites, involving two different hosts and often even different host tissues, they had to find means for efficient adaptation of their metabolism at highly different environments encountered. This implied the development of mechanisms to regulate differentially the expression of their metabolism in different life cycle stages. Thus metabolic flexibility must have been a highly selective advantage during the different stages of this evolutionary scenario. Trypanosomatids apparently have a considerable number of plants-like traits and several plants-like genes encoding homologs of proteins found in either chloroplast or the cytosol of plants and algae. In fact, elegant studies had proved earlier that many of the genes in different Trypanosomatids are orthologues [26]. Thus* Leishmania,* a Trypanosomatid parasite belonging to the order Kinetoplastida, together with Euglenoids, is under Euglenozoa. In this backdrop, thus, it is not surprising to observe that* Leishmania* possesses an efficient sucrose transporter and metabolizing machinery. Recent report on intracellular invertase BfrA, of* L. major* [27], further supports our hypothesis on the participation of intracellular sucrase of* L. donovani* promastigotes in the sucrose metabolism pathway. Our limited findings are a partial endeavor to explore this very intriguing field of metabolite. Concluding remarks can only be that future knowledge of the role of this sucrase enzyme in the physiology and life cycle of the parasite can lead to the opening up of many avenues towards the better perception of parasitic biology and hence containment of this dreaded disease.

## Figures and Tables

**Table 1:** Purification of intracellular sucrase: the table shows the list of different steps, taken during the purification process. The total enzyme activity, the amount of protein recovered, and the specific activity of the sucrase enzyme were estimated from each step of the purification procedure to calculate the increased fold of purification.

Steps of purification	Total activity nmoles of glucose formed/min	Total protein *µ*g	Specific activity nmoles of glucose/mg/min	Fold purification
Crude extract	2084	790002	26.37	1
Size-exclusion chromatography	1075	11078	97.03	3.67
IEC-CM-cellulose	854.36	3628	235.54	8.93
IEC-DEAE Sephadex	459.08	1250	367	13.92
SEC-Sephacryl S-200	154.64	34.54	4477.12	169.78
Hydrophobic chromatography (HA batch absorption)	103.86	11.816	8789.77	333.32
